# Smoking as a correlate of suicidal behavior and self-harm in adolescents with depressive disorders

**DOI:** 10.3389/fpsyt.2026.1859568

**Published:** 2026-07-01

**Authors:** Xiaoxia Wu, Lei Ding, Jiaquan Liang, Lijuan Gao

**Affiliations:** 1 Nanhai Public Health Hospital of Foshan, Foshan, Guangdong, China; 2 Department of Psychiatry, The Third People’s Hospital of Foshan, Foshan, Guangdong, China

**Keywords:** adolescent, depressive disorder, self-injurious behavior, smoking, suicide

## Abstract

Suicidal behavior and self-harm are major public health concerns among adolescents, particularly those with depressive disorders. While smoking has been linked to suicidality in general populations, its independent role in both suicidal behavior and self-harm within well-characterized clinical samples of youth with depressive disorders remains understudied. This study consecutively enrolled 2,343 adolescents (aged 12–18 years) diagnosed with unipolar depression, bipolar disorder, or depressive episode according to DSM-5 criteria at Nanhai Public Health Hospital of Foshan City and The Third People’s Hospital of Foshan between January 2025 and December 2025. Suicidal behavior (≥1 suicide attempt) and self-harm (intentional self-injury or poisoning regardless of intent) were assessed via clinical interviews. Multivariable binary logistic regression models were constructed to identify factors independently associated with each outcome, adjusting for age, sex, education, income, parental education, psychiatric diagnosis, family history of mental illness, physical disease, and smoking status. Among 2,343 participants (mean age 14.99 ± 1.65 years; 77.8% female), the prevalence of self-harm was 76.0% and suicidal behavior was 44.2%. In fully adjusted models, smoking status was the only variable significantly associated with suicidal behavior after adjustment for measured covariates: current smokers (OR = 2.74, 95% CI: 1.81–4.22, P<0.001) and past quitters (OR = 2.32, 95% CI: 1.66–3.28, P<0.001) had higher odds compared to never smokers. For self-harm, current smoking was significantly associated with increased risk (OR = 2.31, 95% CI: 1.33–4.37, P = 0.006). Education level showed a borderline association with self-harm, with each additional year of schooling corresponding to a 10% lower odds (OR = 0.90, 95% CI: 0.81–1.00, P = 0.050); however, this finding should be interpreted cautiously given the exploratory nature of the analysis. No other demographic or clinical variables, including age, sex, or psychiatric diagnosis, were independently associated with either outcome. Smoking was strongly associated with both suicidal behavior and self−harm after adjustment for measured demographic and clinical covariates. These findings underscore the importance of assessing tobacco use as a potential clinical marker of vulnerability in youth mental health settings.

## Introduction

Suicidal behavior and non-suicidal self-injury among adolescents represent a pressing global public health concern (1). Suicide is the fourth leading cause of death in 15–29-year-olds worldwide, and self-harm is one of the strongest predictors of future suicide attempts (2, 3). The prevalence of these behaviors is particularly elevated in clinical populations, with mood disorders such as unipolar and bipolar depression being among the most robust psychiatric risk factors (4, 5). Understanding the specific correlates and correlates for these behaviors within this high-risk demographic is critical for developing targeted prevention and intervention strategies.

While the link between depressive disorders and suicidal/self-harm behaviors is well-established, the factors that differentiate those who engage in these behaviors from those who do not within this clinical population are less clear (6, 7). Traditional sociodemographic factors, such as age, sex, and socioeconomic status, have shown inconsistent associations across studies (8, 9). Consequently, research has increasingly focused on potentially modifiable behavioral correlates that could serve as clinical markers of vulnerability.

A growing body of evidence links smoking to suicidal thoughts and behaviors. Studies in adult psychiatric patients have reported elevated odds of suicide attempts among smokers after adjustment for some confounders (10), and psychological autopsy studies indicate that smoking may amplify suicide risk in certain diagnostic subgroups (11). However, longitudinal research in major depressive disorder suggests that the association may be confounded by shared vulnerabilities factors such as impulsivity and substance use (12, 13). Critically, most of this evidence comes from adult or mixed−age cohorts (14, 15), and large−scale investigations specifically examining smoking as a correlate of both suicidal behavior and self−harm in rigorously characterized adolescent depressive samples are scarce (16). Therefore, the present study aimed to address this gap.

A comprehensive understanding of suicidal behavior and self-harm in adolescents requires a multi-level conceptual framework that integrates individual, familial, school, and societal determinants (17, 18). At the individual level, severity of depressive and anxiety symptoms, impulsivity, aggression, and use of multiple substances (alcohol, cannabis, electronic cigarettes) are robustly linked to suicidality and self-injury. Early adversity, including childhood maltreatment, further elevates risk. Familial factors—parental psychopathology, family history of suicidal behavior, secondhand smoke exposure, and socioeconomic disadvantage—shape vulnerability, while school-related stressors such as academic pressure, bullying, and peer relationships are common proximal precipitants. Broader cultural attitudes toward suicide and the availability of tobacco control and mental health policies also modulate outcomes. Within this complex architecture, cigarette smoking may function as an observable, easily assessed behavioral marker that partially captures a latent profile characterized by impulsivity, emotion dysregulation, and other unmeasured vulnerabilities—such as comorbid alcohol and illicit drug use, personality pathology, and childhood adversity—rather than acting as a direct causal factor. Although the present study is unable to incorporate all these domains, this framework guided our selection of available covariates and the interpretation of our findings.

Therefore, the primary aim of this study was to investigate the prevalence and clinical correlates of suicidal behavior and self-harm in a large cohort of adolescents diagnosed with depressive disorders. Specifically, we sought to determine whether smoking status (current or past) was independently associated with these outcomes after rigorously adjusting for a comprehensive range of demographic and clinical confounders, including psychiatric diagnosis, family history, and socioeconomic factors. Based on previous literature, we hypothesized that smoking would be a significant and significantly associated with for both suicidal behavior and self-harm in this vulnerable population.

## Methods

### Study design and participants

This study was conducted among adolescents diagnosed with depressive disorders at Nanhai Public Health Hospital of Foshan city and The Third People’s Hospital of Foshan between January 2025 and December 2025. A total of 2,343 participants were consecutively enrolled. Inclusion criteria were: (1) age between 12 and 18 years; (2) a clinical diagnosis of unipolar depression (major depressive disorder), bipolar disorder, or other depressive disorder (e.g., other specified depressive disorder, depressive episode not otherwise specified) according to DSM−5 criteria; and (3) completion of all baseline assessments. Participants with severe cognitive impairment, intellectual disability, or acute medical conditions that precluded reliable assessment were excluded. The study protocol was conducted in accordance with the Declaration of Helsinki and approved by the Institutional Review Board of Nanhai Public Health Hospital of Foshan city, and written informed consent was obtained from all participants and their legal guardians. A total of 2,487 eligible patients were approached during the study period, of whom 144 (5.8%) declined participation or had incomplete baseline assessments, yielding a final sample of 2,343 participants and a response rate of 94.2%.

### Measures and data collection

Demographic and clinical characteristics were collected through standardized questionnaires and electronic medical records. Demographic variables included age (years), sex (male/female), education level (years of schooling), annual household income (graded from 1 to 10, with higher scores indicating greater income), father’s education level (graded from 1 to 6, representing increasing educational attainment from illiteracy/primary school to postgraduate education), and mother’s education level (graded from 1 to 6, representing increasing educational attainment from illiteracy/primary school to postgraduate education).

Self−harm behavior was defined broadly as any lifetime intentional self−injury or self−poisoning, irrespective of the individual’s reported suicidal intent at the time. Therefore, suicide attempts involving self−injury or poisoning were also classified as self−harm events. Suicidal behavior was defined as having made at least one suicide attempt in the individual’s lifetime (an act with some non−zero intent to die), regardless of method. Consequently, these two outcome variables are not mutually exclusive: a participant whose suicide attempt involved cutting or overdose would be positive for both self−harm and suicidal behavior, whereas a participant whose only attempt was by hanging would be positive for suicidal behavior but negative for self−harm. Differentiation between non−suicidal self−injury and suicide attempts was made on the basis of the individual’s reported intent at the time of the act, using standardized probes from the assessment protocol (“At the time you did this, did you want to die? Did you think this would kill you?”). Only acts accompanied by a non−zero desire to die were classified as suicidal behavior. The full assessment protocol is provided in **Supplementary File**.

Suicide-related variables were assessed based on clinical interviews. A standardized clinical assessment protocol, developed for this study, was used to evaluate suicidal ideation and behavior (**Supplementary File**). All clinical interviews were conducted face to face by trained psychiatric clinicians (psychiatrists or senior psychiatric nurses) using a structured assessment protocol. To enhance inter rater reliability, interviewers participated in a two-day training workshop that included practice interviews and consensus ratings on pilot cases. Periodic fidelity checks were performed throughout the data collection period.

Suicidal ideation/behavior was categorized into three mutually exclusive groups: none (no lifetime suicidal ideation or behavior), ideation only (lifetime suicidal thoughts with no suicide attempt), and suicidal behavior (≥1 lifetime suicide attempt, defined as a self−injurious act with some non−zero intent to die). Current smokers were defined as individuals who reported smoking at least one cigarette per day for the past month. Past quitters were defined as individuals who had previously smoked daily for at least one month but reported no smoking in the past month. Of note, this assessment was specific to combustible cigarette smoking and did not capture the use of electronic cigarettes (vaping), heated tobacco products, or other nicotine delivery systems, which have become increasingly prevalent among contemporary youth.

### Statistical analysis

Continuous variables were presented as mean ± standard deviation (SD), and categorical variables were presented as frequencies and percentages. For group comparisons, participants were stratified by the presence or absence of suicidal behavior and self-harm behavior. Differences between groups were assessed using the independent samples t-test for continuous variables and the chi-square test for categorical variables. For categorical variables with more than two levels, an overall chi-square test was performed.

For continuous variables, normality was assessed using the Shapiro–Wilk test, and homogeneity of variances was examined with Levene’s test. When the assumptions of normality or equal variances were violated, the Mann–Whitney U test was applied instead of the independent samples t-test. In the present analysis, all continuous variables exhibited non-normal distributions; therefore, group comparisons for these variables were conducted using the Mann–Whitney U test. To identify factors independently associated with suicidal behavior and self-harm behavior, two separate multivariable binary logistic regression models were constructed. All variables listed in the respective tables were entered into the models as covariates to adjust for potential confounders, including age, sex, education, income, parental education, diagnosis, family history of mental illness, physical disease, and smoking status. Results were reported as odds ratios (ORs) with 95% confidence intervals (CIs). Model fit was evaluated using the Akaike Information Criterion (AIC) and McFadden’s pseudo R^2^. Multicollinearity among covariates was assessed using variance inflation factor (VIF) scores, with values <5 considered acceptable. As a sensitivity analysis, we re-ran both models excluding the variable “received treatment for self-harm” (to evaluate possible collider bias) and confirmed that the pattern of associations remained unchanged.

All statistical tests were two-tailed, and a P-value < 0.05 was considered statistically significant. Statistical analyses were performed using R version 4.1.0 (R Foundation for Statistical Computing, Vienna, Austria).

## Results

### Baseline characteristics of the study population

A total of 2,343 participants were included in this study. The mean age of the cohort was 14.99 ± 1.65 years, and the majority were female (77.8%). The most common diagnosis was unipolar depression (83.1%), followed by bipolar disorder (15.2%). The prevalence of self-harm behavior was 76.0%, and suicidal behavior was reported in 44.2% of the participants. The prevalence of suicidal behavior was 43.5% in unipolar depression, 47.9% in bipolar disorder, and 47.4% in depressive episode. Among females, 45.5% reported suicidal behavior, compared with 39.8% of males. Detailed stratified prevalence data for both suicidal behavior and self-harm are presented in **Supplementary Table 1**. The majority of the participants were never smokers (89.2%). Detailed baseline demographic and clinical characteristics are presented in **Table 1**.

**Table 1:** Baseline characteristics of the study population.

Variable	Level	Statistic (%)
Age (years)		14.99 ± 1.65
Sex (male)		517 (22.1)
Education (years)		9.17 ± 1.76
Annual household income (grade)		6.12 ± 2.44
Father’s education level (grade)		2.81 ± 1.30
Mother’s education level (grade)		2.56 ± 1.30
Diagnosis	Unipolar depression	1948 (83.1)
	Bipolar disorder	357 (15.2)
	Depressive episode	38 (1.6)
Family history of mental illness	Yes	220 (9.4)
Physical disease	Yes	65 (2.8)
With self-harm behavior		1780 (76.0)
Received treatment for self-harm		835 (35.6)
Suicidal ideation/behavior	None	147 (6.3)
	Ideation	1161 (49.6)
	Behavior	1035 (44.2)
Number of suicide attempts		6.90 ± 25.04
With suicidal behavior		1035 (44.2)
Smoking status	Never smoker	2089 (89.2)
	Current smoker	102 (4.4)
	Past quitter	152 (6.5)
Daily cigarette consumption (cigarettes/day)		7.26 ± 9.76

Continuous variables are presented as mean ± standard deviation, categorical variables as frequency (percentage).

### Comparisons between groups

As shown in **Table 2**, compared to participants without suicidal behavior, those with suicidal behavior were significantly more likely to have received treatment for self-harm (49.1% vs. 25.0%, P < 0.001) and to be current smokers (6.6% vs. 2.6%) or past quitters (9.2% vs. 4.4%, P < 0.001). No significant differences were observed between the two groups regarding age, sex, education level, household income, or psychiatric diagnosis (all P > 0.05).

**Table 2:** Comparison of characteristics between participants with and without suicidal behavior.

Variable	Level	No suicidal behavior (n=1308)	Suicidal behavior (n=1035)	Statistic	P-value
Age (years)		14.97 ± 1.64	15.02 ± 1.66	-0.67	0.501
Education (years)		9.13 ± 1.71	9.21 ± 1.82	-1.17	0.240
Annual household income (grade)		6.09 ± 2.42	6.17 ± 2.47	-0.75	0.454
Father’s education level (grade)		2.81 ± 1.29	2.81 ± 1.32	0.09	0.929
Mother’s education level (grade)		2.55 ± 1.30	2.57 ± 1.30	-0.45	0.656
Sex, n (%)	Male	293 (22.4)	224 (21.6)	0.15	0.695
	Female	1015 (77.6)	811 (78.4)		
Diagnosis, n (%)	Unipolar depression	1081 (82.6)	867 (83.8)	0.92	0.631
	Bipolar disorder	207 (15.8)	150 (14.5)		
	Depressive episode	20 (1.5)	18 (1.7)		
Family history of mental illness, n (%)	Yes	111 (8.5)	109 (10.5)	2.61	0.106
	No	1197 (91.5)	926 (89.5)		
Physical disease, n (%)	Yes	41 (3.1)	24 (2.3)	1.14	0.285
	No	1267 (96.9)	1011 (97.7)		
Received treatment for self-harm, n (%)	No	981 (75.0)	527 (50.9)	145.04	<0.001
	Yes	327 (25.0)	508 (49.1)		
Smoking status, n (%)	Never smoker	1217 (93.0)	872 (84.3)	46.63	<0.001
	Current smoker	34 (2.6)	68 (6.6)		
	Past quitter	57 (4.4)	95 (9.2)		

Continuous variables are presented as mean ± standard deviation; categorical variables as frequency (percentage). The chi-square test for each categorical variable is an overall test.

Similarly, participants with self-harm behavior showed a significantly higher prevalence of receiving treatment for self-harm (P < 0.001) and a different distribution of smoking status compared to those without self-harm (P = 0.011). The rate of current smokers was 5.0% in the self-harm group versus 2.3% in the non-self-harm group. No significant differences were observed between the two groups in the number of suicide attempts (P = 0.502) or daily cigarette consumption (P = 0.058), although both variables exhibited highly skewed distributions. Other demographic and clinical variables also did not differ significantly between groups (**Table 3**).

**Table 3:** Comparison of characteristics between participants with and without self-harm behavior.

Variable	Level	No self-harm (n=563)	Self-harm (n=1780)	Statistic	P-value
Age (years)		15.05 ± 1.68	14.97 ± 1.64	0.93	0.353
Education (years)		9.28 ± 1.73	9.13 ± 1.76	1.74	0.082
Annual household income (grade)		6.12 ± 2.42	6.12 ± 2.45	-0.02	0.985
Father’s education level (grade)		2.82 ± 1.30	2.81 ± 1.30	0.23	0.818
Mother’s education level (grade)		2.53 ± 1.31	2.57 ± 1.30	-0.68	0.494
Number of suicide attempts		8.17 ± 40.82	6.50 ± 17.25	0.67	0.502
Daily cigarette consumption (cigarettes/day)		11.40 ± 14.71	5.95 ± 7.07	1.97	0.058
Sex, n (%)	Male	125 (22.2)	392 (22.0)	0.00	0.957
	Female	438 (77.8)	1388 (78.0)		
Diagnosis, n (%)	Unipolar depression	462 (82.1)	1486 (83.5)	1.79	0.404
	Bipolar disorder	94 (16.7)	263 (14.8)		
	Depressive episode	7 (1.2)	31 (1.7)		
Family history of mental illness, n (%)	Yes	49 (8.7)	171 (9.6)	0.31	0.580
	No	514 (91.3)	1609 (90.4)		
Physical disease, n (%)	Yes	16 (2.8)	49 (2.8)	0.00	0.958
	No	547 (97.2)	1731 (97.2)		
Received treatment for self-harm, n (%)	No	563 (100.0)	945 (53.1)	408.30	<0.001
	Yes	0 (0.0)	835 (46.9)		
Smoking status, n (%)	Never smoker	519 (92.2)	1570 (88.2)	8.97	0.011
	Current smoker	13 (2.3)	89 (5.0)		
	Past quitter	31 (5.5)	121 (6.8)		

Continuous variables are presented as mean ± standard deviation; categorical variables as frequency (percentage). The chi-square test for each categorical variable is an overall test. The “no self-harm” group includes participants who may have had suicide attempts by methods other than self-injury or poisoning (hanging, drowning), as these acts were not captured by the self-harm definition employed in this study. Consequently, the number of suicide attempts variable is presented for both groups.

### Factors associated with suicidal behavior

To identify factors independently associated with suicidal behavior, a multivariable logistic regression analysis was performed (**Table 4**). All VIF scores were below 1.5, indicating no significant multicollinearity. The sensitivity analysis yielded substantively identical results. After adjusting for all covariates, smoking status emerged as the only variable independently associated with suicidal behavior. Compared to never smokers, current smokers had a significantly higher odds of suicidal behavior (OR = 2.74, 95% CI: 1.81–4.22, P < 0.001), as did past quitters (OR = 2.32, 95% CI: 1.66–3.28, P < 0.001). Other variables, including age, sex, education, income, and psychiatric diagnosis, were not significantly associated with suicidal behavior in the multivariable model (all P > 0.05). The model had a McFadden’s pseudo R^2^ of 0.17.

**Table 4:** Multivariable logistic regression for suicidal behavior.

Variable	Level	β	SE	OR (95% CI)	P-value
Age (years)		-0.020	0.049	0.98 (0.89–1.08)	0.735
Sex	Male			1.00 (reference)	
	Female	0.068	0.102	1.07 (0.88–1.31)	0.504
Education (years)		0.039	0.047	1.04 (0.95–1.14)	0.413
Annual household income (grade)		0.010	0.018	1.01 (0.98–1.05)	0.487
Father’s education level (grade)		-0.030	0.045	0.97 (0.89–1.06)	0.529
Mother’s education level (grade)		0.030	0.047	1.03 (0.94–1.13)	0.538
Diagnosis	Unipolar depression			1.00 (reference)	
	Bipolar disorder	-0.128	0.118	0.88 (0.70–1.11)	0.294
	Depressive episode	0.104	0.335	1.11 (0.57–2.14)	0.754
Family history of mental illness	No			1.00 (reference)	
	Yes	0.239	0.137	1.27 (0.93–1.64)	0.144
Physical disease	No			1.00 (reference)	
	Yes	-0.315	0.263	0.73 (0.43–1.22)	0.238
Smoking status	Never smoker			1.00 (reference)	
	Current smoker	1.008	0.218	2.74 (1.81–4.22)	<0.001
	Past quitter	0.842	0.175	2.32 (1.66–3.28)	<0.001
Model statistics	N			2343	
	AIC			3185.5	
	Pseudo R²			0.17	

β, regression coefficient (log odds); SE, standard error; OR, odds ratio; CI, confidence interval. Pseudo R² is McFadden’s pseudo R².

### Factors associated with self-harm behavior

A separate multivariable logistic regression was conducted to examine factors associated with self-harm behavior (**Table 5**). Consistent with the findings for suicidal behavior, current smoking was significantly associated with an increased odds of self-harm (OR = 2.31, 95% CI: 1.33–4.37, P = 0.006). Additionally, education level showed a marginally significant protective effect; each additional year of education was associated with a 10% lower risk of self-harm (OR = 0.90, 95% CI: 0.81–1.00, P = 0.050). No other variables, including age, sex, diagnosis, or family history, were independently associated with self-harm behavior (all P > 0.05). The model’s McFadden’s pseudo R^2^ was 0.15.

**Table 5:** Multivariable logistic regression for self-harm behavior.

Variable	Level	β	SE	OR (95% CI)	P-value
Age (years)		0.068	0.058	1.07 (0.96–1.20)	0.232
Sex	Male			1.00 (reference)	
	Female	0.010	0.117	1.01 (0.80–1.27)	0.955
Education (years)		-0.105	0.054	0.90 (0.81–1.00)	0.050
Annual household income (grade)		0.000	0.020	1.00 (0.96–1.04)	0.992
Father’s education level (grade)		-0.051	0.054	0.95 (0.86–1.06)	0.367
Mother’s education level (grade)		0.068	0.051	1.07 (0.96–1.18)	0.239
Diagnosis	Unipolar depression			1.00 (reference)	
	Bipolar disorder	-0.139	0.133	0.87 (0.67–1.13)	0.282
	Depressive episode	0.329	0.454	1.39 (0.64–3.47)	0.440
Family history of mental illness	No			1.00 (reference)	
	Yes	0.104	0.173	1.11 (0.80–1.56)	0.539
Physical disease	No			1.00 (reference)	
	Yes	-0.030	0.306	0.97 (0.56–1.78)	0.919
Smoking status	Never smoker			1.00 (reference)	
	Current smoker	0.837	0.324	2.31 (1.33–4.37)	0.006
	Past quitter	0.270	0.214	1.31 (0.88–2.00)	0.195
Model statistics	N			2343	
	AIC			3215.5	
	Pseudo R²			0.15	

β, regression coefficient (log odds); SE, standard error; OR, odds ratio; CI, confidence interval. Pseudo R² is McFadden’s pseudo R².

## Discussion

This study of 2,343 adolescents with depressive disorders investigated the prevalence and clinical correlates of suicidal behavior and self-harm. The main findings reveal alarmingly high rates of both phenomena, with 44.2% of participants reporting a history of suicidal behavior and 76.0% reporting self-harm. After rigorous multivariable adjustment, smoking status emerged as the sole independently associated with for suicidal behavior and a significant correlate of self-harm. Specifically, current smokers and past quitters had approximately 2.5 times higher odds of suicidal behavior compared to never smokers, while current smokers had more than double the odds of engaging in self-harm. A marginally significant protective effect was observed for higher education level against self-harm.

The prevalence of self−harm (76.0%) and suicidal behavior (44.2%) in our cohort is high, which can be attributed to several converging factors. First, the sample was recruited exclusively from regional specialist psychiatric hospitals, which concentrate youth with greater illness severity and acute risk. Second, we used systematic, face−to−face clinical interviews specifically designed to probe these behaviors, a method known to yield higher disclosure than unstructured clinical notes or self−report scales. Third, the inclusion of both unipolar and bipolar depression, the latter being associated with greater impulsivity, may elevate base rates. Fourth, our lifetime assessment captures events that may predate the current treatment episode. Our rates are at the upper end of those reported in meta−analyses of adolescent psychiatric inpatients (19). These findings support the interpretation that this sample represents a particularly high−severity, help−seeking population, and the prevalence figures should not be generalized to community settings.

Our primary finding is that smoking status was the only variable that remained significantly associated with both suicidal behavior and self-harm after adjustment for the measured covariates. This association persisted even after controlling for available demographic and clinical confounders, including psychiatric diagnosis, family history, and socioeconomic factors. This association persisted even after controlling for available demographic and clinical confounders, including psychiatric diagnosis, family history, and socioeconomic factors. The observation that both current and past smokers exhibited elevated odds is consistent with a potential cumulative or persistent vulnerability, although formal dose-response testing was not conducted. This aligns with a growing body of evidence linking smoking to suicidal thoughts and behaviors in both general population and psychiatric cohorts (14, 20). The most clinically prudent interpretation is that smoking, in this adolescent psychiatric population, is not a direct cause of suicidal or self-harm behaviors, but rather a robust proxy for a broader constellation of unmeasured vulnerabilities. Nicotine dependence frequently co-occurs with higher trait impulsivity, difficulties in emotion regulation, a propensity for externalizing behaviors, and exposure to adverse childhood environments—all of which are potent associated with suicidality and self-harm (21, 22). In our models, which did not include measures of these psychological constructs, smoking status may have served as a statistical surrogate for this latent risk profile. From a neurobiological perspective, it has been hypothesized that smoking and nicotine dependence are associated with dysregulation of neurotransmitter systems, particularly serotonin, which is also implicated in the pathophysiology of depression and impulsivity (23, 24); however, given the cross-sectional and correlational nature of our data, these mechanistic hypotheses remain speculative and require direct testing in prospective designs. Chronic smoking can also lead to significant physical health problems, which may, in turn, exacerbate feelings of hopelessness and despair (25). The finding that past quitters also had a significantly elevated odds is noteworthy, but rather than implying a lasting neurobiological consequence of prior smoking, this pattern more likely reflects that a history of regular smoking severe enough to prompt a quit attempt is a marker of a more severe premorbid phenotype—characterized, for example, by higher baseline impulsivity—which itself confers persistent risk. The modest McFadden pseudo−R² values (0.15–0.17) indicate that the variables in our models explain only a small fraction of the variance in suicidal and self−harm behaviors. This underscores the multifactorial nature of these outcomes and aligns with our interpretation that smoking likely functions as a marker for a broader, unmeasured risk profile. Interestingly, the univariate comparison showed that the mean number of suicide attempts was numerically higher in the non-self-harm group, but this difference was not statistically significant and was driven by extreme outliers, as indicated by the large standard deviation. This underscores the importance of multivariable adjustment and the use of robust statistical methods when analyzing skewed data.

Consistent with the conceptual framework outlined in the Introduction, the modest McFadden pseudo−R² values (0.15–0.17) indicate that the measured variables explain only a small fraction of the variance in suicidal and self−harm behaviors. This is in line with the well-recognized multifactorial nature of these outcomes, where accurate prediction requires comprehensive assessment across individual, familial, and environmental domains (26). Our inability to measure key constructs such as depressive symptom severity, impulsivity, and other substance use likely accounts for the limited explanatory power and underscores the need for caution in causal interpretation.

Interestingly, the association between smoking and self-harm, while significant, was less pronounced than for suicidal behavior. This may suggest that different, though overlapping, pathways are involved. Self-harm can serve various functions, including emotion regulation and self-punishment, and may be driven more by acute distress than the long-term neurobiological consequences of chronic smoking (27, 28). The marginally significant protective effect of education on self-harm warrants mention. Education may confer resilience through enhanced problem-solving skills, greater cognitive flexibility, better future orientation, and access to a supportive social network (3). It may also serve as a proxy for higher cognitive function and socioeconomic advantage, which are known to be protective against a range of adverse mental health outcomes. Contrary to some previous studies, we did not find significant independent associations between suicidal or self-harm behaviors and variables such as age, sex, or specific psychiatric diagnosis within this clinical sample (29, 30). The absence of independent associations for established factors previously reported to be associated female sex, older age, and bipolar diagnosis may reflect the unique composition of this clinical sample. Given that the cohort consisted exclusively of treatment-seeking adolescents with depressive disorders, the restricted range of illness severity and demographic homogeneity may have attenuated the ability to detect these effects. Additionally, the high overall prevalence of suicidal behavior and self-harm in this sample suggests that participants may represent a particularly high-severity subgroup of youth with mood disorders, potentially limiting the generalizability of these null findings to less severely affected populations. It is possible that in a severely affected clinical population, traditional demographic correlates are overshadowed by more proximal behavioral ones.

## Limitations

First, the cross−sectional design precludes any causal inference. While we identified smoking as a correlate, we cannot determine whether smoking preceded the outcomes, followed them, or both resulted from shared underlying vulnerabilities. Second, the data were collected from two public health hospitals in a single geographic region, which may limit the generalizability of the findings to other settings or populations. Third, key variables, including smoking history and suicidal/self-harm behaviors, were based on self-report and clinical interviews, which are subject to recall and social desirability biases. Fourth, and perhaps most critically, our models could not adjust for several important domains highlighted by the conceptual framework, including severity of depressive and anxiety symptoms, trait impulsivity, aggression, history of childhood trauma, alcohol and illicit drug use, electronic cigarette use, secondhand smoke exposure, family history of suicidal behavior, school-related stressors (academic performance, bullying), and broader socio-cultural variables. The absence of these variables likely contributes to the modest model fit and raises the possibility of unmeasured confounding. Consequently, the observed associations should not be interpreted as evidence of a direct causal effect of smoking; rather, smoking may serve as a readily observable marker for a constellation of unmeasured vulnerabilities that are themselves associated with suicidal and self-harm behaviors. Future prospective studies incorporating comprehensive assessments across these domains are needed to delineate specific pathways. Fifth, because the self-harm definition included suicide attempts by self-injury/poisoning, the self-harm group is heterogeneous, comprising both non-suicidal self-injury and some suicide attempts. This limits the ability to draw conclusions specific to NSSI alone. The non-significant difference in mean number of suicide attempts between the self-harm and non-self-harm groups may be an artifact of this classification, as the non-self-harm group contains individuals who attempted suicide by other methods. Sixth, the assessment of smoking was limited to combustible cigarettes and did not capture duration, intensity, or use of electronic cigarettes (vaping) or other nicotine delivery systems. Given the rising popularity of vaping among adolescents, our findings cannot reflect the full spectrum of nicotine use and its associated risks. Seventh, outcomes were based on lifetime recall, which may be subject to memory biases and does not capture the timing or acuity of risk. The intensity of suicidal behavior (number of attempts) was recorded but, due to extreme skew, could not be examined as an outcome; future work should explore whether smoking differentially relates to single versus multiple attempts. Finally, the model fit indices were low, suggesting that the variables included in our models explain only a small portion of the variance in these complex behaviors. Furthermore, as this was a hospital-based study conducted in a specialized mental health setting, selection bias may limit generalizability to community-dwelling adolescents with mood disorders. Participants seeking care at a public health hospital may differ systematically from those who do not, potentially exhibiting greater illness severity or different patterns of health behavior. In addition, the reliance on self-report and clinical interview for ascertaining smoking status and self-harm introduces the possibility of information bias, including underreporting due to social desirability or stigma. While we attempted to mitigate this by using standardized assessment protocols, the absence of biochemical verification of smoking or collateral reports may have led to misclassification of exposure and outcome.

## Conclusions

In this clinical sample of adolescents with depressive disorders, smoking was strongly associated with both suicidal behavior and self−harm after adjustment for the measured demographic and clinical covariates. However, the cross−sectional design and the absence of key variables such as impulsivity, substance use, and trauma history preclude any causal interpretation. Smoking status likely serves as a readily observable clinical marker of a broader, high−risk phenotype. Mental health professionals should therefore routinely assess tobacco use as part of a comprehensive psychosocial evaluation, while recognizing that effective prevention requires attention to the multiple shared vulnerabilities that underlie both smoking and self−injurious behaviors.

## Data Availability

The original contributions presented in the study are included in the article/**Supplementary Material**. Further inquiries can be directed to the corresponding authors.
