# Supplementary material for: Smoking as a correlate of suicidal behavior and self-harm in adolescents with depressive disorders
Source: Front Psychiatry. 2026 Jul 1;17:1859568. doi: 10.3389/fpsyt.2026.1859568 (PMC13369496; doi:10.3389/fpsyt.2026.1859568)
Supplement: Supplementary file 1 [file DataSheet1.pdf]

Clinical Assessment Protocol for Suicidal Behavior, Self-Harm, and Smoking Status

1. Self-Harm Behavior

Definition: Intentional self-injury or poisoning, regardless of the presence of suicidal intent.

Assessment Questions:

1. Have you ever deliberately hurt yourself on purpose (for example, by cutting, burning, hitting, or scratching your skin) without the intention to die?

   - Yes / No

   - If yes: How many times in the past year? ____ times; in your lifetime? ____ times.

2. Have you ever deliberately taken more medication than prescribed, or taken any substance, with the intention to harm yourself even if you did not intend to die?

   - Yes / No

   - If yes: How many times? ____

3. (For participants answering yes to either question) Did you receive any medical or psychiatric treatment for these self-harm behaviors?

   - Yes / No

Coding: Participants answering "yes" to question 1 and/or 2 were classified as having a history of self-harm behavior.

2. Suicidal Behavior

Definition: At least one lifetime suicide attempt (an intentional act with at least some intent to die).

Assessment Questions:

1. Have you ever had thoughts about ending your life (suicidal thoughts)?

   Never / In the past year / More than one year ago

2. Have you ever made a suicide attempt (an actual, intentional act to end your life)?

   Yes / No

   If yes: How many attempts in your lifetime? ____

   If yes: When was the most recent attempt? (Within past month / 1–12 months ago / More than 1 year ago)

2a. (For participants who answered “yes” to question 2) Differentiation of intent:

“At the time you did this, did you want to die?” (Yes / No)

“Did you think this act would kill you?” (Yes / No)

(Only acts accompanied by a non-zero desire to die were classified as suicidal behavior. Acts without suicidal intent were classified as self-harm only.)

3. For participants with multiple attempts:

   - “Would you describe the most serious attempt as something that required medical attention?”

     Yes / No

Coding: Participants answering “yes” to question 2 and reporting at least some intent to die in question 2a were classified as having a history of suicidal behavior.

3. For participants with multiple attempts:

- “Would you describe the most serious attempt as something that required medical attention?”

- Yes / No

Coding: Participants answering “yes” to question 2 were classified as having a history of suicidal behavior.

3. Smoking Status

Assessment Questions:

1. Have you ever smoked a cigarette, even one or two puffs?

- Yes / No →If No, classify as “Never smoker” and skip remaining questions.

2. Do you currently smoke cigarettes (at least one cigarette per day) or have you smoked in the past?

- I smoke daily now

- I smoke occasionally now (less than daily)

- I used to smoke, but I have quit completely

3. For current smokers:

- On average, how many cigarettes do you smoke per day? ____ cigarettes/day

4. For past quitters:

- How long ago did you stop smoking? (Weeks / Months / Years)

- Before quitting, how many cigarettes did you usually smoke per day? ____ cigarettes/day

Coding:

- Never smoker: Answered “No” to ever having smoked.

- Current smoker: Currently smokes daily or occasionally.

- Past quitter: Used to smoke but has now quit completely.

Note: All assessments were conducted as part of a structured clinical interview at baseline. The protocol was developed for this study to ensure consistency across participants. Responses were recorded in the electronic medical record and later extracted for analysis.
