# Supplementary material for: Smoking as a correlate of suicidal behavior and self-harm in adolescents with depressive disorders
Source: Front Psychiatry. 2026 Jul 1;17:1859568. doi: 10.3389/fpsyt.2026.1859568 (PMC13369496; doi:10.3389/fpsyt.2026.1859568)
Supplement: Supplementary file 2 [file Table1.docx]

**Supplementary Table S1.** Prevalence of suicidal behavior and self‑harm by diagnosis and sex.

| Stratification | Category | Total n | Suicidal behavior, n (%) | Self‑harm, n (%) |
| --- | --- | --- | --- | --- |
| Diagnosis | Unipolar depression | 1948 | 847 (43.5) | 1486 (76.3) |
|  | Bipolar disorder | 357 | 171 (47.9) | 263 (73.7) |
|  | Depressive episode | 38 | 18 (47.4) | 31 (81.6) |
| Sex | Female | 1826 | 830 (45.5) | 1388 (76.0) |
|  | Male | 517 | 205 (39.8) | 392 (75.8) |
